# Retraction Note: A pH/enzyme dual responsive PMB spatiotemporal release hydrogel promoting chronic wound repair

**DOI:** 10.1186/s12951-024-02960-0

**Published:** 2024-11-05

**Authors:** Lanlan Dong, Can Huang, Baohua Zhao, Guangyun Hu, Yong Huang, Xiaorong Zhang, Xiaohong Hu, Ying Wang, XiaoyanSun, Wei Qian, Gaoxing Luo

**Affiliations:** 1 College of Bioengineering, Chongqing University, Chongqing, 400044 People’s Republic of China; 2 Institute of Burn Research, Southwest Hospital, Third Military Medical University (Army Medical University), Chongqing, 400038 People’s Republic of China; 3 Department of Medical Innovation, Research Center for Wound Healing & Regenerative Medicine, Chinese PLA General Hospital, Beijing, 100853 People’s Republic of China

**Retraction Note: Journal of Nanobiotechnology (2023) 21:213**

10.1186/s12951-023-01947-7

The authors have retracted this article. After publication, the authors identified two overlapping image panels in Fig. 8a: the Day 1 image panels for Groups 1 and 5 overlap, and the Day 3 image panel for Group 3 overlaps with the Day 3 image panel for the 0 mg/ml condition in Fig. 3a. A review of the raw data for these figures after these concerns were identified highlighted a further case of overlap between two images representing different experimental conditions. The authors therefore no longer have confidence in the reliability of the data presented in this article.

Wei Qian and Gaoxing Luo have stated on behalf of all authors that they agree with this retraction.

